# Lupus-like oral mucosal lesions in mercury-induced autoimmune response in Brown Norway rats

**DOI:** 10.1186/1471-2172-14-47

**Published:** 2013-10-03

**Authors:** Kei Seno, Jun Ohno, Nobutaka Ota, Takao Hirofuji, Kunihisa Taniguchi

**Affiliations:** 1 Department of General Dentistry, Division of General Dentistry, Fukuoka Dental College, 2-15-1 Tamura, Fukuoka, Japan; 2 Department of Morphological Biology, Division of Pathology, Fukuoka Dental College, 2-15-1 Tamura, Fukuoka, Japan; 3 Department of Oral and Maxillofacial Surgery, Division of Oral Oncology, Fukuoka Dental College, 2-15-1 Tamura, Fukuoka, Japan

**Keywords:** Mercury-induced autoimmunity, Oral mucosa, Lupus-like lesions, Brown Norway rats

## Abstract

**Background:**

Administration of mercury at nontoxic doses induces systemic autoimmune disease in Brown Norway (BN) rats. The pathogenesis of lupus-like oral mucosal lesion by mercury-induced autoimmunity is still unclear, even though the oral mucosa is observed to be commonly affected in mercury-treated BN rats. In this study, we investigated the immunopathology of lupus-like oral mucosal lesions in a model of mercury-induced systemic autoimmunity.

**Methods:**

Brown Norway male rats were injected subcutaneously with either phosphate-buffered saline (control) or mercury at a dose of 1.0 mg per kilogram of body weight on days 0, 3, 5, and 7. Blood, kidney, and tongue samples were taken at various timepoints for evaluation by immunohistochemistry, RT-PCR, and lupus band test (LBT).

**Results:**

Oral mucosal lesions were classified according to three consecutive temporal phases on the basis of infiltration of immunocompetent cells as follows: (phase I) infiltration of MHC class II^+^ dendritic cells (DC) and macrophages; (phase II) addition of ED1^+^ macrophage infiltrates; and (phase III) focal infiltration of pan T cells following increased infiltration of DC and macrophages. Dense infiltration of DC and macrophages was observed in the basement membrane (BM) zone of the oral epithelium. Tissue expression of IL-4 mRNA was detected in early lesions (phase I), suggesting that locally produced IL-4 may be responsible for Th2-mediated immune response. A linear and continuous smooth pattern of fluorescence was observed in the oral epithelial BM in addition to renal glomeruli, indicating immune complex deposits.

**Conclusions:**

Local autoimmune responses are involved in the pathogenesis of mercury-induced lupus-like lesions of the oral mucosa.

## Background

The etiology of autoimmune diseases remains largely unclear despite numerous research efforts, including clinical studies, epidemiological studies, and those involving experimental models. Hypothetical concepts speculate that autoimmunity results from a susceptible genetic background and the impact of specific environmental factors, including infectious agents and chemicals/xenobiotics [1-4]. Therefore, autoimmune diseases seem to develop because of environmental triggers in combination with genetic and stochastic factors.

Induction of autoimmune disease by environmental agents, especially mercury, in susceptible rodent strains is a well-established and relevant model of systemic autoimmunity [5,6]. In Brown Norway (BN) rats, repeated administration of nontoxic doses of mercury chloride (HgCl_2_) leads to T cell-dependent polyclonal activation of B cells characterized by lymphoproliferation, hypergammaglobulinemia, production of autoantibodies and immune complex (IC) deposits in the renal glomerular mesangium [7,8]. Target organs of systemic mercury-induced autoimmunity include lymphoid organs, kidneys, salivary glands, and mucocutaneous tissues [7]. Among the mucocutaneous tissues, the oral mucosa is a target of mercury in BN rats [7,9,10]. Histopathological findings of mercury-induced oral mucosal lesions were characterized by dense infiltration of mononuclear cells, including dendritic cells (DC) and macrophages, in the lamina propria beneath the surface epithelium of the mucosa. These changes seem to result from the establishment of mercury-induced autoimmunity in the oral mucosa. However, there are few research studies of mercury-induced disease that show evidence of direct interaction between local autoimmunity and cell infiltrates of immunocompetent cells in the oral mucosa.

Lupus erythematosus (LE) is a chronic inflammatory condition, considered the prototype of autoimmune human disease. Classically, LE has been subdivided into systemic and cutaneous forms. Whereas systemic LE is a multiorgan disease with variable prognoses, cutaneous LE is a more benign condition, limited to the skin and/or mucosal surfaces [11]. The prevalence of oral mucosal involvement in LE patients is debatable. Some authors suggest that oral lesions are present in 9% - 45% patient with systemic LE and 3% -20% in those with cutaneous LE [11]. These clinical data prompted us to examine whether systemic autoimmunity can lead to established lupus lesions in the oral mucosa. In the present study, we examined oral mucosal lesions in BN rats with systemic mercury-induced autoimmunity. First, we identified that immunohistopathological findings are characterized by dense infiltration of DC and macrophages in the basement membrane (BM) zone. Second, we determined that tissue expression of IL-4 mRNA is detected from early phase of oral lesions. Finally, the lupus band test is positive at junction between epithelium and lamina propria. Collectively, our data demonstrate that systemic mercury-induced autoimmunity induces LE-like lesions in the oral mucosa.

## Methods

### Rats

Inbred adult male BN rats (RTl^n^) weighing 250–350 g were purchased from Kyudo Co. (Saga, Japan). All experiments were performed using rats matched for strain, age, and gender. Male rats were used because of their greater susceptibility to mercury-induced autoimmunity [12]. The animal experimentation protocols were approved by the Animal Care and Use Committee of Fukuoka Dental College.

### HgCl_2_ treatment

Groups of rats were injected with phosphate-buffered saline (PBS) or HgCl_2_ (Sigma-Aldrich, St. Louis, MO, U.S.A.). Mercury-induced autoimmunity in the BN rats has been established by Aten et al. [7]. HgCl_2_, dissolved in distilled water (1 mg/ml), was injected at a dose of 1.0 mg of mercury per kilogram of body weight subcutaneously at days 0, 3, 5, and 7. At least five animals were used for each experimental point. PBS-treated rats were used as controls in all experiments.

### Blood and tissue preparation

Blood samples were collected from tail veins of the mercury treated and control rats at various time points. The serum was separated and stored at −80°C for serum autoantibody determination. Kidneys and tongues were excised 2, 4, 6, 8, 10, 12, 14, and 21 days after injection from the mercury-treated group (n=5 each). Control tissues were collected from the control group at each time point (n=3 each). Half of the tissue specimens were fixed in 4% paraformaldehyde in PBS and embedded in paraffin. Paraffin sections (4-μm thickness) were then stained with hematoxylin and eosin (HE) to help visualize histopathological changes. The other specimens were immediately frozen in liquid nitrogen, and serial frozen sections were used for immunostaining, immunofluorescence (IF), and extraction of total RNA.

### Immunohistochemistry

Monoclonal antibodies (mAb) used for immunohistochemistry are listed in Table 1. Acetone-fixed frozen sections were first incubated with normal rabbit serum to decrease nonspecific binding and then reacted with one of the mAbs. Sections were incubated with alkaline phosphatase-conjugated anti-mouse antibody (1:150 dilution; DakoCytomation, Tokyo, Japan). Immunohistochemical reactions were visualized using 5-bromo-4-chloro-3-indolyl phosphate/nitro blue tetrazolium chloride solution (BCIP/NBT solution; DakoCytomation). As a control, sections were treated with normal mouse IgG instead of the first set of antibodies. Infiltrating cells in the epithelial and subepithelial regions of the oral mucosa were counted in 25 randomly selected areas of 50 μm^2^ each. Statistical analysis was performed with the two-tailed Student’s *t*-test. Data are presented as the mean ± standard error and *P* values of < 0.05 were considered statistically significant.

**Table 1:** Monoclonal anti-rat monoclonal antibodies used for immunohistochemical analysis

Antibody (mAb) *	**Epitope**	**Specificities**
OX6	RT1B	MHC class II antigens
OX19	CD5	Pan T cells
ED1	CD68	Macrophages, monocytes

*All mAbs were purchased from Cedarlane Laboratory, Burlington, ON, Canada.

### Real-time reverse transcription-polymerase chain reaction (qRT-PCR)

Total RNA was isolated from serial frozen sections by acid guanidiniumthiocyanate-phenol-chloroform extraction using an ISOGEN Kit (Nippon Gene, Toyama, Japan). One microgram of total RNA was transcribed into cDNA using random primers, oligo (dT) primers, and 10 μl of reverse transcriptase (ReverTra® Ace qPCR RT Kit; Toyobo Co., Ltd., Osaka, Japan). The reverse transcription was performed at 37°C for 15 min and then at 98°C for 5 min. Resulting templates were subjected to a LightCycler Nano real-time PCR system according to the manufacturer’s procedure (Roche Diagnostics, Tokyo, Japan). Predesigned primers and probe reagents for rat interleukin-4 (IL-4), interferon-γ (IFN-γ), and glyceraldehyde-3-phosphate dehydrogenase (G3PDH) were commercially obtained from Roche Diagnostics. G3PDH was used as an internal control. The relative quantification of mRNA expression was calculated as a ratio of IL-4 and IFN-γ genes to G3PDH. Sequences of the primers and TaqMan probe were as follows: IL-4 forward primer, 5′-CATCGGCATTTTGAACGAG-3′; reverse primer, 5′-CGAGCTCACTCTCTGTGGTG-3′; Universal ProbeLibrary probe no. 2; IFN-γ forward primer, 5′-TCAAAAGAGTTCCTTATGTGCCTA-3′; reverse primer, 5′-TACGAGGACGGAGAGCTGTT-3′; Universal ProbeLibrary probe no. 69; G3PDH forward primer, 5′-AATGATCCGTTGTGGATCTGA-3′; reverse primer, 5′-GCTTCACCACCTTCTTGATGT-3′; Universal ProbeLibrary probe no. 80. Furthermore, 1.8% agarose gels were run to confirm that clean products of the expected length had been obtained.

### Detection of autoantibodies and lupus band test (LBT) by IF

Serum samples from the mercury-treated or control rats were tested for the presence of autoantibodies by indirect IF. Detection of antinuclear autoantibodies (ANA) was used for HEp-2 cells as substrate [13]. Briefly, serum samples were diluted 1:50–1:1000 in PBS and were incubated on slides with monolayer HEp-2 cells (GA Generic Assay, Dahlewitz, Germany), followed by Alexa Flour 488-conjugated goat anti-rat IgG Ab (Molecular Probes, Eugene, OR, USA) diluted 1:100. Titers were expressed as the reciprocal value of the highest serum dilution that gave a clear positive reaction. No staining at a serum dilution of 1:50 was considered as a negative result. *In situ* binding of anti-BM autoantibodies from serum samples was tested by indirect IF using frozen sections of the kidneys and tongues from the control rats as substrates [14]. Serum samples diluted 1:50 up to 1:1000 were incubated on frozen sections of kidneys and tongues. Binding sites of serum samples were detected by Alexa Flour 488-conjugated goat anti-rat IgG (Molecular Probes). The titers were expressed as the reciprocal value of the highest serum dilution that gave a clear positive reaction.

LBT was performed by the direct IF method. Frozen sections of the kidneys and tongues from the mercury-treated or control rats were incubated with FITC-conjugated mouse anti-rat IgG, Fcγ (Jackson ImmunoResearch Laboratories, West Grove, PA, USA), diluted 1:10 up to 1:500.

## Results

### Immunohistochemical staging of mercury-induced oral mucosal lesions by mononuclear cell infiltrates

**Figure 1:**
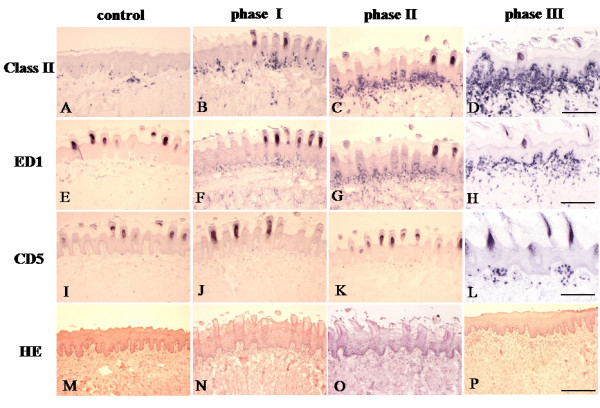
**Immunohistochemical staging of infiltrating mononuclear cells in the tongue of the mercury-treated rats. A**-**D**: The tongue specimens from the control rats show MHC class II^+^ cells distributed sparsely to both the lamina propria and surface epithelium **(A)**. The number of MHC class II^+^ cells increases continuously as phase progress **(B**-**D)**. **E**-**H**: A few ED1^+^ cells are in the lamina propria of normal tongue **(E)**. During progression of phases, the number of ED1^+^ cells is increased in upper lamina propria **(F**-**H)**. **I**-**L**: A small number of CD5^+^ T cells are distributed in the lamina propria of tongue specimens from phases I and II as well as the control rats **(I-K)**. In phase III specimens, focal accumulations of CD5^+^ T cells are present in both the lamina propria and surface epithelium **(L)**. **M**-**P**: No obvious changes are noted in hematoxylin-eosin (HE)-stained tongue sections taken from thec ontrol, phase I and phase II rats **(M**-**O)**. Infiltrates of mononuclear cells are observed in the lamina propria of the oral mucosa in phase III specimens **(P)**. Bar= 100μm.

**Figure 2:**
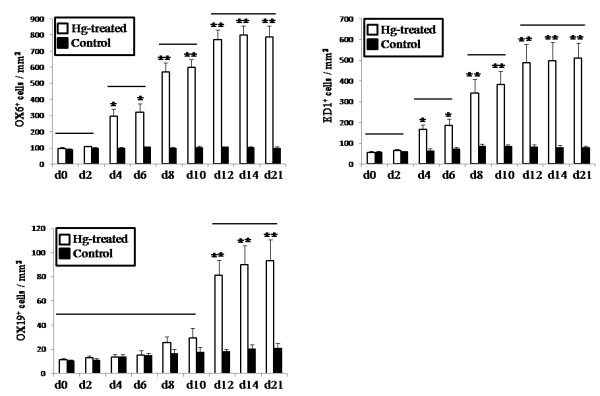
**Infiltration of MHC class II**^**+**^**, ED1**^**+**^**, and OX19**^**+ **^**mononuclear cells in the tongue from the control rats and the mercury-treated rats.** Cells were counted from day 0 (d0) to day 21 (d21). The tongues from control rats are represented by *black columns* (n=3 per day), the tongues from mercury-treated rats are indicated by *white columns* (n=5 per day). Cell numbers/ mm^2^ indicated as mean± standard error (SE). *, significantly different at *p*< 0.01 compared with control. **, significantly different at *p*< 0.005 compared with control. There are no significant differences in groups of *white columns* jointed to horizontal bars are at a *p* value of <0.05.

**Figure 3:**
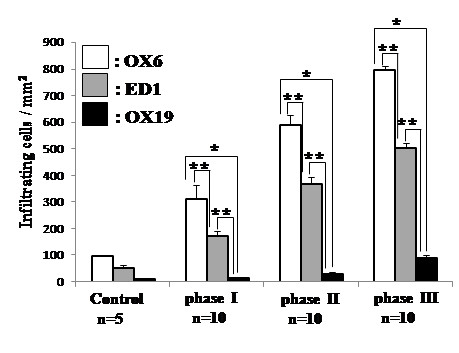
**The number of MHC class II**^**+**^**, ED1**^**+**^**, and OX19**^**+ **^**mononuclear cells at various phases of oral mucosal lesions in the mercury-treated rats.** Cell numbers/ mm^2^ indicated as mean± standard error (SE). *, significantly different at *p*< 0.01. **, significantly different at *p*< 0.05.

We first analyzed infiltration of the oral mucosa from the control and mercury-treated rats by MHC class II^+^, ED1^+^, and CD5^+^ cells and classified the oral mucosal lesions according to three consecutive phases on the basis of density and distribution of infiltrating cells (Figures 1, 2 and 3). In this study, two different subpopulations of macrophages were detected with monoclonal antibodies OX 6 and ED1 [15,16]. Anti-OX 6 antibodies, which bind to antigen-presenting cells, have been conventionally used to detect both DCs and macrophages in normal and pathological tissue sections from rats. Positive DCs in particularly are thought to function as antigen-presenting cells. Anti-ED1 antibody is a conventional marker for macrophages of rats and binds to a CD68-like intracellular antigen. To compare infiltrates of the macrophage system with those of pan T cells, the CD5 antigen, was detected with anti-OX19 antibody.

### Untreated control tongues

In the tongue of the control rats, MHC class II was expressed by Langerhans cells, DCs, and macrophages in the surface epithelium and lamina propria of the oral mucosa (Figure 1A). ED1^+^ macrophages were detected sparsely in the lamina propria and submucosal tissue (Figure 1E). Only a few T cells were observed in the lamina propria (Figure 1I). No histological changes were seen with HE staining (Figure 1M).

### Phase I (days 4–6)

Two days after mercury administration, the number of infiltrating cells in the tongue showed no significant difference in the mercury-treated rats compared with that in the control rats (Figure 2). The first phase comprised days 4–6 after the subcutaneous injection of mercury in the rats and was characterized by an increased infiltration of MHC class II^+^ and ED1^+^ mononuclear cells (Figures 1B and 1F, respectively). The number of cells positive for both markers was significantly higher in the mercury-treated rats than in the control rats (*p*< 0.001; Figure 2). Furthermore, increased number of cells positive for both markers in this phase was also significantly higher than that from mercury-treated rats by day 2 ( *p*< 0.05; Figure 2). The rate of increase in the number of ED1^+^ cells was much less than that of MHC class II^+^ cells (*p*< 0.05; Figure 3). Most MHC class II^+^ cells showed a dendritic-shape. Both MHC class II^+^ and ED1^+^ cells were mainly localized in the lamina propria beneath the surface epithelium of the mucosa. In contrast, the density of CD5^+^ T cells and the HE staining pattern in the mercury-treated rats closely resembled that in control rats (Figures 1J and 1N). The number of T cells in the tongue was not significantly different between the mercury-treated rats and the control rats (Figure 2) and it less than that of MHC class II^+^ DCs or ED1^+^ macrophages (*p*< 0.01; Figure 3).

### Phase II (days 8–10)

A phase II of oral mucosal lesions in the mercury-treated rats at 8–10 days showed the continued increment of infiltrates of MHC class II ^+^ and ED 1^+^ cells in the lamina propria (Figures 1C and 1G, respectively). Both MHC class II^+^ and ED1^+^ cells tended to accumulate in the BM zone. The number of MHC class II^+^ and ED1^+^ cells increased higher in phase II than that of those cells in phase I (*P*< 0.05; Figure 2). However, the density of MHC class II^+^ cells was significantly high, compared with that of ED1^+^ cells (*P*< 0.05; Figure 3). Infiltrates of T cells and tongue histology by HE staining remained unchanged (Figures 1K and 1O). The number of T cells in phase II showed no significant difference, compared with that in phase I (Figures 2).

### Phase III (days 12 and later)

Infiltrates of CD5^+^ T cells occurred during the third phase, which started after day 12. The number of T cells was higher (*p*< 0.05) in this phase than in phase II (Figure 2). Focal infiltrates of T cells were noted in the lamina propria of the oral mucosa (Figure 1L). The continued increase in the number of MHC class II^+^ and ED1^+^ cells remained in this phase, showing a significant difference at *p*< 0.05, compared with phase II (Figure 2). In this phase, massive infiltration of MHC class II^+^ DCs showed a tendency to attach to the surface epithelium (Figure 1D). Similarly, ED1^+^ macrophages accumulated in the epithelial BM zone of the tongue (Figure 1H). Among these infiltrating cells, the number of MHC class II^+^ cells was highest, like other phases (Figure 3). HE staining revealed cellular infiltrates in the lamina propria of the oral mucosa, but epithelial degeneration was not found (Figure 1P). After day 21, the density of cell infiltrates gradually decreased.

### IL-4 mRNA expression in the oral mucosa of the mercury-treated rats

**Figure 4:**
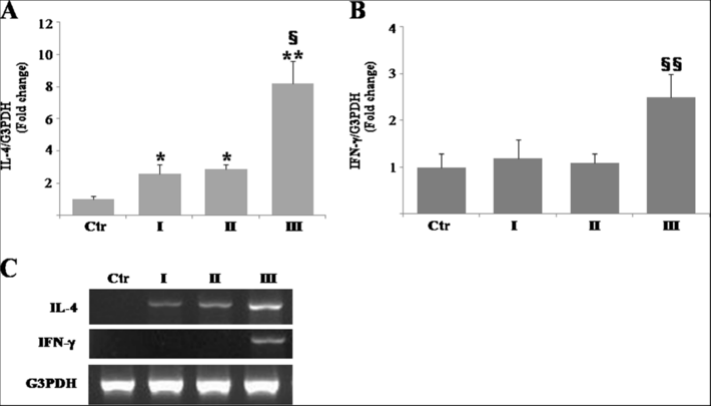
**Tissue expression of interleukin-4 (IL-4) and interferon-γ (IFN-γ) mRNA in the tongue of the mercury-treated rats by real-time reverse transcription-polymerase chain reaction (qRT-PCR). A** and **B**: qRT-PCR analyses examine expression levels of mRNA encoding IL-4 **(A)** and IFN-γ **(B)** normalized by those of glyceraldehyde-3-phosphate dehydrogenase (G3PDH). Data represents mean ± standard error (SE) of pooled data derived from three to five independent experiments. *, significantly different at *p*< 0.05 compared with the control. **, significantly different at *p*<0.01 compared with the control. §, significantly different at *p*<0.05 compared with phases I and II. §§, significantly different at *p*<0.05 compared with control, phases I, and II. **C**: Agarose gel electrophoresis analysis of qRT-PCR. Ctr, control; I, phase I; II, phase II; III, phase III.

In the mercury-treated rats, a Th2-dominated autoimmune response is induced in target organs [17]. We therefore examined tissue expression of IL-4 (Th2) and IFN-γ (Th1) mRNA in the tongue during each phase of the study. qRT-PCR analyses indicated that expression levels of mRNA encoding IL-4 normalized by that of G3PDH increased in phases I and II by 2.6 fold and 2.9 fold when compared with their control, respectively, and reached the maximum in phase III by 8.2 fold (Figure 4A). In contrast, expression levels of IFN-γ mRNA remained unchanged until phase II, whereas those increased in phase III by 2.5 fold when compared with the control (Figure 4B). These data from qRT-PCR analyses were identical to those of agarose gel electrophoresis analyses (Figure 4C).

### Presence of autoantibodies in serum samples of the mercury-treated rats

**Figure 5:**
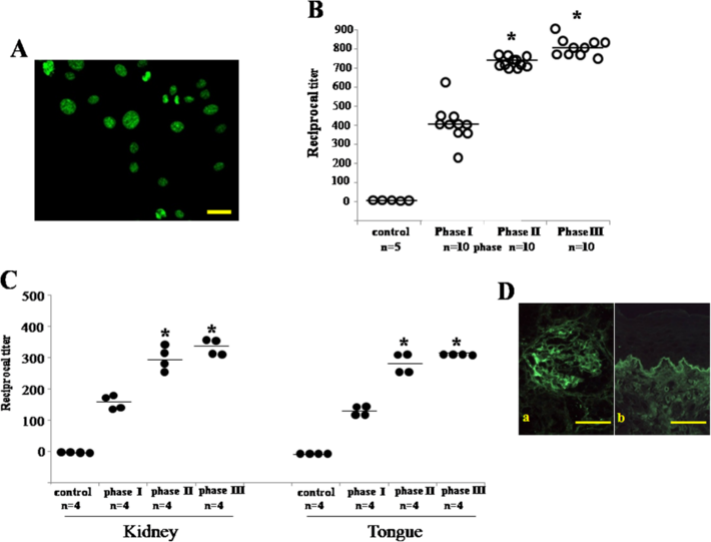
**Antibody immunofluorescence (IF) analysis. A**: Detection of antinuclear autoantibodies (ANA) by indirect IF using serum from the mercury-treated rats with phase III lesions, incubated on HEp-2 cells. Both nucleolar and nuclear cytoplasmic fluorescences are noted. Bar=50 μm. **B**: The reciprocal titer of IgG ANA in test serum samples from the rats in control and various phases. Horizontal bars denote median values. *, significantly different at *p*<0.05 compared with phase I. Controls show no titers. **C**: The reciprocal titer of autoantibodies bound to renal capillaries and basement membrane (BM) of the oral mucosa by indirect IF analysis. Horizontal bars denote median values. *, significantly different at *p*<0.05 compared with phases I. Controls show no titers. **D**: Indirect IF images of autoantibodies in the renal capillaries and basement membrane (BM) of the oral mucosa using serum from Brown Norway (BN) rats with phase III lesions. **(A)** IF reaction of the renal capillaries and mesangium is seen in the normal kidney. **(B)** IF labeling on the BM of the oral epithelium in normal tongue. Bars= 100 μm.

Autoimmune lesions resulting from exposure to mercury in mice are characterized by elevated levels of serum autoantibodies, including ANA and antibody to glomerular capillaries in kidney [13,18]. We performed ANA detection using HEp-2 cells. Serum samples, diluted 1:50, from the control rats barely bound to the HEp-2 cells. These cells remained negative when reacted with serum samples from the mercury-treated rats by day 4. Binding of serum samples, taken from the mercury-treated rats in phase I, was detected as combined nucleolar and nuclear cytoplasmic fluorescence in HEp-2 cells (Figure 5A). Figure 5B shows changes in serum titers during each phase of the study. The serum titer was undetectable in the control rats. After mercury-treatment, the titers increased with progression of the lesions. The serum titer in the mercury-treated rats was 400±67.87 in phase I , and it increased to 783.33±89.67 in phase II. In phase III, IgG ANA titer eventually reached 800±78.67. Reciprocal serum titer of IgG ANA in both phase II and III was significantly higher than that in phase I (*p*< 0.01) (Figure 5B).

We next performed indirect IF staining of frozen sections from the control rat kidney and tongue specimens with serum samples from the mercury-treated rats to determine whether those serum samples could bind to glomerular capillaries and the BM zone of the oral mucosa. Comparison of endpoint titers in indirect IF staining were summarized in Figure 5C. Staining with serum was absent in the kidney and tongue in control rats, showing undetectable levels of antibodies. Similar to the ANA assay, the titer increased during progression of lesion. The serum titer of renal and lingual deposits was significantly (*p*<0.05) higher in phases II and III compared with phase I (Figure 5C). Serum samples from phase III rats bound fluorescently to the glomerular capillaries and mesangium of frozen kidney sections (Figure 5Da). Similarly, these serum samples reacted with the epithelial BM of the oral mucosa from the tongue of the control rats (Figure 5Db).

### LBT in kidney and tongue specimens from the mercury-treated rats

**Figure 6:**
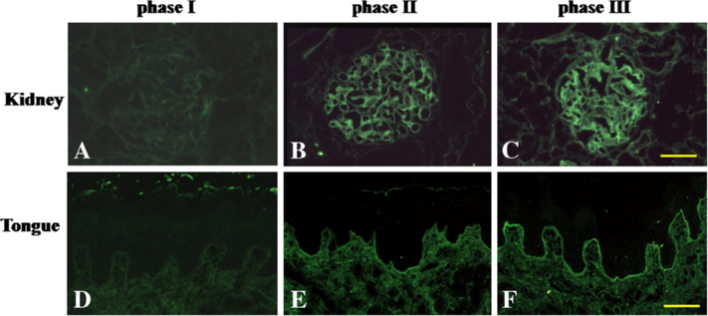
**Lupus band test (LBT) with FITC-conjugated anti-rat IgG, Fcγ, antibody (1:300) incubated on frozen sections of the kidneys and tongues from mercury-treated rats. A**-**C**: No immunofluorescence (IF) staining is seen in phase I **(A)**. IF reaction on the glomerular mesangial regions during phases II **(B)** and III **(C)**. **D**-**F**: A very weak or faint reaction is seen in the portion of the basement membrane (BM) of the oral epithelium of the tongue by phase I **(D)**. Remarkable IF appears on the BM of the epithelium of the tongue in phases II **(E)** and III **(F)**. Bar= 100 μm.

Mercury-induced autoimmunity is also characterized by the appearance of IC, mostly in the kidneys [19-22]. We therefore examined kidney and tongue tissue specimens by LBT during each phase of the study. In this study, LBT was performed using the highest dilution (1:300) of anti-rat IgG antibody to examine the severity of IgG deposits between phases in the development of mercury-induced oral mucosal lesions. The endpoint antibody titer was decided by preliminary experiments (data not shown). Renal tissues from the control and phase I rats showed no IgG deposits (Figure 6A); however, intense, linear, continuous smooth deposits of IgG were observed by IF in the glomerular capillary walls and mesangium of kidneys during phases II - III in the mercury-treated rats (Figures 6B and 6C). In the tongues from the control and phase I rats, no deposits of IgG were found (Figure 6D). In contrast, an obvious deposition of IgG was detected in the epithelial BM of the oral mucosa from both phase II and III rats (Figures 6E and 6F). Reactive IF showed a linear and continuous smooth pattern. These results suggested that the severity of immune deposits on the kidney and tongue strengthened in phase II and III compared with phase I.

## Discussion

In this study we used BN rats treated subcutaneously with mercury to elucidate whether oral mucosal lesions develop as a result of local events associated with immune responses, which were similar to those in systemic LE. Although the oral mucosa has been proposed as one of the targets in systemic autoimmune disorders of rats administered mercury, uncertainty remains as to whether the pathology underlying oral mucosal lesions include local immunological phenomena [7,9,10,23,24]. We present three lines of evidence to support the conclusion that local immunological events play a role in the elicitation of lupus-like oral mucosal lesions an accompanying symptom of systemic mercury-induced autoimmunity. First, immunohistochemical approaches confirmed that an infiltration of both DC and macrophages occurred early during lesion development. Second, RT-PCR results indicated that local IL-4 release was found in the early lesions. Third, IgG deposition was detected at the junction between the surface epithelium and lamina propria of the oral mucosa by LBT.

The immunohistochemical results presented here indicate that infiltration of DCs and macrophages occurs in the early stage of oral lesion formation related to systemic mercury-induced autoimmunity. These findings are supported by previous reports [9,10,24]. Because infiltration of DCs and macrophages precedes that of T cells, we speculate that development of oral lesions is mediated by DCs and macrophages. Generally, increased MHC class II antigen is most often associated with immunological stimuli [25]. The earliest events in which MHC class II^+^ cells infiltrate around the BM zones suggest that local immune responses related to systemic mercury-induced autoimmunity may be established in the oral mucosa. Following increased infiltration of MHC class II^+^ cells, numerous ED1^+^ macrophages migrate to the BM zone, indicating that the BM zone is the target tissue of oral mucosal lesions in systemic mercury-induced autoimmunity. Furthermore, accumulation of macrophages can indicate phagocytosis of injured fragments around the BM zone. Although increased infiltration of DCs and macrophages is not a primary event in mercury-induced autoimmunity, it may play an important secondary role in the pathogenesis of this syndrome. In contrast to the predominance of DC/macrophage-infiltration, participation of T cells seem to be less important in oral lesions. These findings suggest that DCs and macrophages may accumulate to react against local immune responses around the BM zone.

RT-PCR showed that tissue expression of IL-4 mRNA was observed from the early phase of oral lesions, suggesting that a local Th2-mediated immune response is responsible for the development of mercury-induced oral mucosal lesions. Previous RT-PCR analyses of mercury-treated BN rats show upregulation of IL-4 mRNA [26,27]. These reports speculate that IL-4 may serve multiple roles in the development of lupus. Phase I specimens were found to express IL-4 mRNA by RT-PCR, along with immunohistochemically detected infiltration of DC and macrophages in the absence of T cells. These findings suggest that cells other than T cells may be associated with IL-4 synthesis, even though T cells are known to participate in the secretion of this cytokine [28,29]. Although our immunohistochemical results reported here do not identify the type (s) of cells expressing IL-4 mRNA, a previous study demonstrated that mercury induced upregulation of IL-4 mRNA expression in mast cells of BN rats [27]. Future work will address the origin of cells bearing IL-4 mRNA in mercury-induced oral mucosal lesions.

The observation of upregulated expression of IFN-γ mRNA in phase III specimens was unexpected. IFN-γ is generally responsible for the development of Th1-mediated immune responses, such as graft-versus-host disease. Our previous report examined IFN-γ induced epithelial expression of intercellular adhesion-1 (ICAM-1) in the early phase of oral mucosal graft-versus-host disease [30]. It is difficult to explain why IFN-γ expression is upregulated in the late stage of mercury-induced oral mucosal lesions. Mercury-induced autoimmunity in BN rats is generally characterized by its weak symptoms, most of which resolve by 20 days after onset [31]. A speculative possibility is that a Th2-mediated immune response responsible for the development of mercury-induced oral lesions may be decreased by the predominant Th1-mediated immune response in the late stage.

IF analyses indicate that the oral mucosa is one of the targets in mercury-induced systemic autoimmunity in BN rats. According to autoantibody production results and a positive LBT reaction in renal glomeruli, we support the mercury-treated BN rats as a model for the development of a systemic lupus-like syndrome. These findings are in concordance with data from other research groups, which showed that the features of mercury-induced autoimmunity in rats and mice, including lymphadenopathy, hypergammaglobulinemia, humoral autoimmunity, and IC deposits, are consistent with the autoimmunity observed in systemic LE [21,32-34]. In this study, serum samples from the mercury-treated BN rats contained autoantibodies, such as ANA and anti-BM antibody. It is known that ANAs could signal the body to begin attacking itself, which can lead to autoimmune diseases, including lupus, scleroderma, Sjögren’s syndrome, drug-induced lupus, and autoimmune hepatitis [35,36]. Indirect IF, using serum samples from the mercury-treated rats, showed a linear reaction in the renal glomeruli. Similarly, LBT of the kidney in the mercury-treated rats revealed a linear IF reaction in the glomeruli. LBT can be used to confirm IC-mediated reactions. Both indirect and direct IF studies demonstrated the deposition of glomerular IC in mercury-treated rat kidneys. Glomerular IC deposits are a hallmark of lupus-like autoimmune disease [22]. In the tongue of the mercury-treated rats, LBT, as well as indirect IF assays, showed the linear deposition of IC in the BM zone, similar to the findings in the kidney. This suggests that the oral mucosa is affected by the lupus-like autoimmune disease. On the basis of these results, we suggest that the lupus-like oral mucosal lesions from mercury-induced systemic autoimmunity, are initiated by the deposition of IC in BM, followed by infiltration of DCs and macrophages into the IC deposition.

In summary, these results provide additional support for the characterization of oral mucosal lesions, in mercury-induced autoimmune disease. The IC deposits in BM are undoubtedly a key step in the pathogenesis of lupus-like oral mucosal lesions.

## Conclusions

Mercury-induced systemic autoimmune responses induce lupus-like lesions in the oral mucosa.

## Abbreviations

ANA: Antinuclear antibody; BM: Basement membrane; BN: Brown Norway; DC: Dendritic cell; G3PDH: Glyceraldehyde-3-phosphate dehydrogenase; HE: Hematoxylin and eosin; HgCl2: Mercury chloride; IC: Immune complex; IF: Immunofluorescence; LBT: Lupus band test; LE: Lupus erythematosus; MAb: Monoclonal antibody; PBS: Phosphate-buffered saline; RT-PCR: Reverse transcription-polymerase chain reaction.

## Competing interests

The authors declare that they have no competing interests.

## Authors’ contributions

KS and JO planned the study, performed the experiments and data analysis, and wrote the manuscript. NO carried out the immunostaining and helped to draft the manuscript. TH and KT supervised manuscript writing. All authors read and approved the final manuscript.
